# Data on small cardamom transcriptome associated with capsule rot disease

**DOI:** 10.1016/j.dib.2019.104625

**Published:** 2019-10-08

**Authors:** K. Mary Mathew, Renjanan Reshma, M. Geethu, Varghese Rithin, K.K. Sabu, F. Nadiya, Muhammad Ali Noushad, Soumya S. Dharan, Y.S. Rao, A.B. Remashree

**Affiliations:** a Indian Cardamom Research Institute (Spices Board Min Commerce & Industry, Government of India), Myladumpara, Idukki, Kerala 685553, India; b Jawaharlal Nehru Tropical Botanic Garden and Research Institute, Palode, Thiruvananthapuram 695562, India

**Keywords:** Small cardamom, RNA sequencing, Transcriptome, Differential expression

## Abstract

Small cardamom (*Elettaria cardamomum* (L.) Maton, also known as the ‘*Queen of Spices*’ is a rhizomatous herbaceous monocot from the family Zingiberaceae. In the present study, using HiSeq™ 2000 RNA sequencing technology, transcriptome sequencing was performed for both control and disease stressed small cardamom leaf tissues. RNA-seq generated 46,931,637 (101 base) and 31,682,496 (101 base) raw reads and totally 9.93GB and 6.63GB of sequence data for cardamom control and stressed samples respectively. The raw data were submitted to NCBI SRA database of under the accession numbers SRX2512359 and SRX2512358 for the control and diseased samples respectively. The raw reads were quality filtered and assembled using TRINITY *de novo* assembler which created 1,11,495 (control) and 91,096 (diseased) contigs with N50 values 3013 (control) and 2729 (stressed). The data was further used to identify significantly differentially expressed unigenes between control and stressed samples. Assembled unigenes were further annotated and evaluated *in silico* to predict the function using publicly available databases and gene annotation tools.

**Table:** Specifications Table

Subject	Agricultural and Biological Sciences	
Specific subject area	Plant Science	
Type of data	Text (FASTQ sequence files), table	
How data were acquired	Data were generated using RNA HiSeq™ 2000 RNA sequencing technology	
Data format	Raw data in FASTQ format	
Parameters for data collection	Freshly collected leaf samples (from both control and infected small cardamom plants) was used for RNA isolation	
Description of data collection	RNA seq libraries representing control and capsule rot disease stressed small cardamom were prepared and transcriptome sequencing was performed and *de novo* assembled to generate unigenes	
Data source location	Plant growth and treatments were given under controlled conditions at ICRI, Idukki, Kerala, India. Data was generated from Illumina HiSeq™ 2000	
Data accessibility	Data is available at NCBI SRA https://www.ncbi.nlm.nih.gov/sra/SRX2512359 https://www.ncbi.nlm.nih.gov/sra/SRX2512358

**Table:** 

**Value of the Data** • Capsule rot disease, commonly known as Azhukal disease is reported to be one of the most serious fungal diseases in small cardamom caused by *Phytophthora meadii* [1] often leading to annual loss of 30–40% [2]. • Under fungal infections R genes and many other defense related genes triggering disease tolerance to plants may get over expressed. • Transcriptome data generated from leaves of plants grown under specific conditions could provide information on molecular mechanism underlying disease tolerance. • Differential expression analysis of control and treated cardamom could compare the expression variation of particular genes in normal and diseased plant grown under similar conditions.

## 1 Data

Data shared in this article includes RNA-seq generated paired end strand specific 46,931,637 (101 base) and 31,682,496 (101 base) raw reads and totally 9.93GB and 6.63GB of sequence data for cardamom control and stressed samples respectively.

## 2 Experimental design, materials, and methods

### 2.1 Plant material

Leaf tissues from both sets, *i.e.*, naturally infected capsule rot and non-infected control plants were collected followed by immediate freezing in liquid nitrogen. Ten biological replicates were pooled from leaf tissues under these two conditions [3].

### 2.2 Total RNA isolation and transcriptome sequencing

RNA extraction was done using a modified protocol of RNeasy Plant Mini Kit (Qiagen) and CTAB method [4]. RNA integrity and quality analysis was done using 2100 BioAnalyzer (Agilent Technologies). Illumina sequencing was performed using the HiSeq™ 2000 platform as per the manufacturer's instructions (Illumina, San Diego, CA). RNA-seq generated paired end strand specific 46,931,637 (101 base) and 31,682,496 (101 base) raw reads and totally 9.93GB and 6.63GB of sequence data for cardamom control and stressed samples respectively.

### 2.3 *De novo* transcriptome assembly and functional annotation

The raw reads were pre-processed to remove adapter sequences, low quality bases, tRNAs and rRNAs. *De novo* transcriptome assembly was performed with TRINITY program [5] to generate the assembled contigs. The assembler created 1,11,495 and 91,096 contigs for control and stressed cardamom samples (Table 1). The assembled unigenes were used for further downstream analysis such as annotation to publicly available databases, Gene Ontology (GO) enrichment and finally validation of differentially expressed genes using qPCR. Additionally, the reads from both pairs were combined and assembled together to generate a reference transcriptome (1,62,589 contigs, 310.7 MB). The information provided by the current study might be useful in developing molecular markers, SNPs, screening of R genes and marker assisted selection to develop superior cultivar varieties in cardamom.

**Table 1:** Read and assembly statistics of control and stressed cardamom data.

Plant Material	Control	Diseased
Total number of raw reads	46,931,637*2 = 93,863,274	31,682,496*2 = 63,364,992
Total number of bases	9.93 GB	6.63 GB
Initial GC%	43	44
Read length	101	101
GC% after trimming	43	43.5
Reads after adapter removal and quality trimming	46,097,664*2 = 92,195,328	31,183,779*2 = 62,367,558
Total contigs	111,495	91,096
Max Contig Length	17,667	16,840
N50	3013	2729
Total Length	243,651,614	185,125,249
GC% after assembly	39.90	40.35
Total size of assembly	274.5 MB	210.6 MB
